# Psychiatric pharmacogenomics predicts health resource utilization of outpatients with anxiety and depression

**DOI:** 10.1038/tp.2013.2

**Published:** 2013-03-19

**Authors:** J Winner, J D Allen, C Anthony Altar, A Spahic-Mihajlovic

**Affiliations:** 1 AssureRx Health, Inc. 6030 S. Mason Montgomery Road, Mason, OH, USA; 2 Department of Psychiatry, University of Illinois at Chicago, Chicago, IL, USA

**Keywords:** cytochrome P450, pharmacoeconomics, pharmacogenomics, psychotropic, medication

## Abstract

Antidepressants are among the most widely prescribed medications, yet only 35–45% of patients achieve remission following an initial antidepressant trial. The financial burden of treatment failures in direct treatment costs, disability claims, decreased productivity, and missed work may, in part, derive from a mismatch between optimal and actual prescribed medications. The present 1 year blinded and retrospective study evaluated eight direct or indirect health care utilization measures for 96 patients with a DSM-IV-TR diagnosis of depressive or anxiety disorder. The eight measures were evaluated in relation to an interpretive pharmacogenomic test and reporting system, designed to predict antidepressant responses based on DNA variations in cytochrome P450 genes (*CYP2D6*, *CYP2C19*, *CYP2C9* and *CYP1A2*), the serotonin transporter gene (*SLC6A4*) and the serotonin 2A receptor gene (*5HTR2A*). All subjects had been prescribed at least one of 26 commonly prescribed antidepressant or antipsychotic medications. Subjects whose medication regimen included a medication identified by the gene-based interpretive report as most problematic for that patient and are in the ‘red bin' (medication status of ‘use with caution and frequent monitoring'), had 69% more total health care visits, 67% more general medical visits, greater than three-fold more medical absence days, and greater than four-fold more disability claims than subjects taking drugs categorized by the report as in the green bin (‘use as directed') or yellow bin (‘use with caution'). There were no correlations between the number of medications taken and any of the eight healthcare utilization measures. These results demonstrate that retrospective psychiatric pharmacogenomic testing can identify past inappropriate medication selection, which led to increased healthcare utilization and cost.

## Introduction

Treatment resistance in patients medicated initially for depression or anxiety is more common than treatment response,^1^ and generates significant financial burden as measured by patient treatment costs, disability claims, decreased productivity and missed work.^2, 3, 4, 5^ An objective method to identify appropriate medications could reduce the incidence of treatment resistance and the increased health care utilization that accompanies failed medication trials. Psychiatric pharmacogenomics, a gene-based method to improve precision in psychotropic medication prescribing, analyzes polymorphisms in pharmacokinetic and pharmacodynamic genes that affect the metabolism of and response to antidepressant and antipsychotic medications.^6^

**Figure 1:**
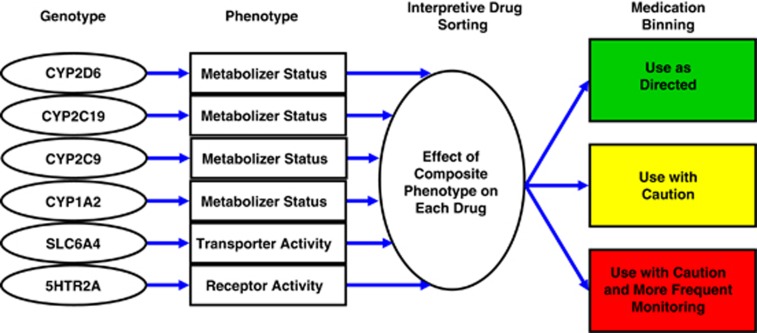
The GeneSight binning method. The patient's genotype is determined for each of the six genes in the panel. A composite phenotype for each drug is created based on the phenotypes predicted from each of the six genotypes. The 18 most commonly prescribed antidepressants and eight antipsychotics on the panel are positioned in a green, yellow or red ‘bin'.

**Figure 2:**
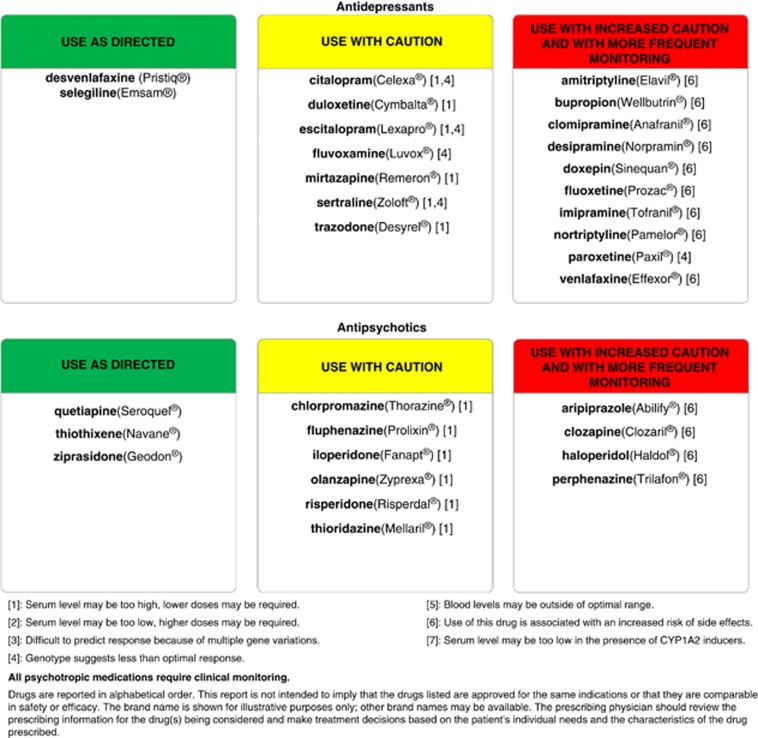
Representative GeneSight medication report based on the genotype of an individual patient.

One example of this approach (Figure 1) shows how combinations of multiple genes and their polymorphisms are analyzed and incorporated into a genotype interpretive report, termed GeneSight (Figure 2), which is available from AssureRx Health, Inc., a personalized medicine company.^7^ The GeneSight interpretive report used in this present study evaluates polymorphic variations in six genes for each individual patient to generate a unique phenotypic profile of drug metabolism and responses for 26 antidepressant and antipsychotic drugs. The approach is designed to assist clinicians in selecting medications for individual patients based on objective, evidence-based genomic information with the goal of improving clinical outcomes in patients with depression and anxiety,^8, 9^ and predicting those drugs that may lead to failed medication trials and poor patient prognoses.^10^

Although healthcare costs associated with negative drug–drug interactions have been studied extensively,^5^ little research exists on the economic impact of pharmacogenomic-based treatment on healthcare resource utilization or the cost burden of inadequate psychiatric medication treatments.^11^ The present retrospective study evaluated whether the GeneSight pharmacogenomic test could predict a variety of direct and indirect healthcare costs and medical utilization for patients with depression and anxiety in a large, multi-specialty, union healthcare system. The unique nature of this healthcare system allowed for the capture of data that are normally difficult to obtain, such as disability claims, missed work and multi-specialty healthcare appointments.

## Materials and methods

The present study was performed at the Union Health Services (UHS), a staff model health maintenance organization located in downtown Chicago, IL, USA. The UHS provides healthcare services for members of collective bargaining service worker unions in the Chicago area. The population is comprised primarily of employed adults and consists of a heterogeneous ethnic mix. The study was approved by Quorum Review, Inc., an independent institutional review board.^12^

The study psychiatrist (AS-M) is a board-certified psychiatrist and the sole contractor for psychiatric care at the UHS. The study population consisted of current psychiatric patients who were consecutively identified and screened by the study psychiatrist, and referred to the study coordinator for informed consent and further screening for inclusion/exclusion criteria. Inclusion criteria for the 97 eligible subjects required a DSM-IV-TR diagnosis of depressive disorder or anxiety disorder, and each patient was being treated with one or more of the 26 medications on the GeneSight medication panel. These drugs are listed in Figure 2, which is a de-identified copy of an actual GeneSight report for one of the subjects included in this study. Exclusion criteria consisted of previous pharmacogenomic testing and diagnoses of bipolar disorder type I, schizophrenia or schizoaffective disorder.

The psychiatric medication regimen for each subject was recorded at the onset of data collection and subject enrollment (1 April 2011), and retrospective healthcare utilization information was obtained to that date beginning on 1 April 2010. The 12-month period was selected to obtain the most current information from subjects seen throughout the same year so that temporally-based prescribing trends would not bias the data (for example, comparing a patient's medication regimen from years other than the 2010–2011 period). This information included the number of outpatient nonpsychiatric medical office visits, outpatient psychiatric office visits, emergency room visits, number of hospitalizations, days of hospitalizations, number of days absent from work and the number of disability claims filed by study subjects. The number and duration of psychiatric and nonpsychiatric medication trials during the year were also recorded.

Following subject consent, a buccal swab was collected by a health professional at the UHS and shipped to the AssureRx Health CLIA-certified and CAP-accredited laboratory in Mason, OH, USA. Following DNA extraction and amplification, variations in 50 alleles were measured for the following six genes: four cytochrome P450 genes (*CYP2D6*, *CYP2C19*, *CYP2C9* and *CYP1A2*), the serotonin transporter gene (*SLC6A4*) and the serotonin 2A receptor gene (*5HTR2A*). Genotype results were converted to a composite phenotype for each psychiatric medication on the panel using the GeneSight interpretive report, in which each of the 26 medications were placed in the category of ‘use as directed' (green bin), ‘use with caution' (yellow bin) or ‘use with caution and more frequent monitoring' (red bin), as exemplified in Figures 1 and 2.

In the primary analysis, the green, yellow or red medication status for each subject was categorized by the highest caution level of any panel medication they were taking at the end of the 1 year observation window. For example, a subject with a CYP2D6 poor metabolizer status and prescribed fluoxetine and mirtazapine would receive a GeneSight report that listed fluoxetine in the red bin and mirtazapine in the yellow bin, reflecting the inherent difference in P450 metabolism for each drug. This patient would be placed in the red bin category owing to their red bin status for fluoxetine. Had the same patient only been on mirtazapine, a yellow bin category would have applied.

The three groups of patients classified by medication status were then compared using a one-way analysis of variance test for differences in each of the eight utilization variables. As a greater number of prescribed drugs would be expected to increase the severity of bin status, we also recorded the number of panel drugs taken concurrently by subjects in each of the three bin groups. The correlations between the mean number of panel drugs, all psychiatric drugs or all drugs in each bin status were determined for each of the eight economic outcomes. Following the one-way analysis of variance, multiple analysis *t*-tests were used to compare pairs of each binned group.

In a secondary analysis, we determined the number of weeks each subject was on a green, yellow or red bin medication during the 1 year study window. For example, out of the 52 weeks, one subject spent 29 weeks on a red bin medication. The time spent on a medication in each bin was correlated with the primary outcomes (healthcare visits, medical visits, psychiatric visits, medical absence days and disability claims) over the 1 year study window. A Pearson's correlation coefficient was calculated as was a *P*-value using the *t*-test.

Average per-event cost estimates for office-based visits, emergency room visits, hospitalizations and days in hospital were obtained using data from the Medical Expenditure Panel Survey database.^13, 14, 15^ The Medical Expenditure Panel Survey database is a set of large-scale surveys of families and individuals, their medical providers and employers across the USA, which was established by the Agency for Healthcare Quality and Research, a division of the US Department of Health and Human Services. Average per-event prescription cost estimates were obtained from data by the Henry J. Kaiser Family Foundation, which were not available in the Medical Expenditure Panel Survey database.^16^ The Kaiser Family Foundation is a nonprofit, nonpartisan organization with a focus on health policy analysis. Average per-event cost estimates for medical absence days and disability claims were obtained using data from Ivanova *et al.*^17^ All cost estimates were multiplied by the number of events, then adjusted to 2010 dollars using a formula of a 3% increase per year to correct for inflation. Costs were compared by the medication bin of subjects using a *t*-test.

## Results

**Figure 3:**
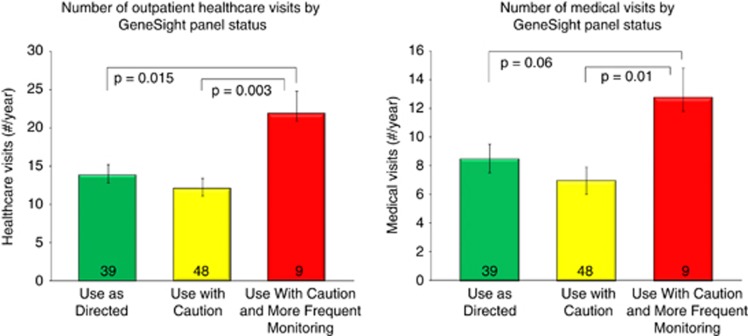
Annual number of healthcare visits (left) and medical visits (right) per patient, by the GeneSight-designated category of subjects' medication regimen.

Of the 97 subjects, nine (9%) were categorized as red bin status, 48 (50%) as yellow bin and 39 (41%) as green bin status based upon the panel medications they were taking at the end of the 1 year observation period. One subject was not on panel medication at the end of the period. Four statistically significant results were obtained when comparing the status of subjects' medication regimens with healthcare utilization histories. Compared with the green or yellow bin subjects, subjects whose regimen included a medication in the red bin had 69% more total healthcare visits (21.9) during the year than those receiving drugs that were in the green bin (13.7) or yellow bin (12.3) (*F*=4.50; df=(2, 93), *P*=0.014). Nonpsychiatric medical visits during the year were 67% higher for subjects whose regimen included a medication in the red bin (12.8) than those on a green (8.4) or yellow bin (7.1) medication (*F*=3.36; df=(2, 93), *P*=0.039) (Figure 3). The relationship between medication bin and number of outpatient psychiatric visits during the study window trended higher for red bin subjects (8.9) than green (5.1; *P*=0.057) or yellow (5; *P*=0.066) bin subjects (*F*=1.97; df=2, 93).

**Figure 4:**
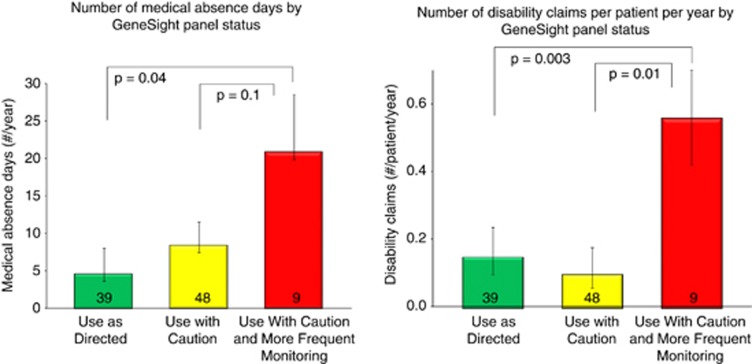
Annual days of medical absences (left) and disability claims (right) per patient, by the GeneSight-designated category of subjects' medication regimen.

Red bin subjects also averaged more disability claims per person during the study period (0.56) than subjects in the green (0.15) or yellow (0.11) bin (*F*=4.54; df=2, 93), (*P*=0.013). Although the overall analysis of variance was suggestive but not significant across all drug bins and number of medical absence days (*P*=0.126), *t*-tests revealed a significant difference between the number of medical absence days between subjects on red and green binned drugs. Red-binned subjects had 20.8 medical absence days, compared with green (4.6), with yellow in-between (8.4) (*P*=0.043, Figure 4). Hospitalizations, days in the hospital and emergency room visits were not significantly different among the groups.

In the secondary analysis, it was observed that the number of weeks subjects spent on red bin medications was correlated with higher total healthcare visits (*r*=+0.56, *P*=0.05). The correlation between number of weeks on a red bin medication and number of disability claims approached significance (*r*=0.5, *P*=0.08). The correlation between number of weeks on a red bin medication and number of medical visits trended in a similar direction (*r*=+0.44, *P*=0.13), as did number of psychiatric visits (*r*=+0.47, *P*=0.10). Number of weeks on a red bin medication was not correlated with numbers of medical absence days, hospitalizations, days in the hospital, or emergency room visits. No statistically significant correlations were observed between number of weeks on a green bin medication or number of weeks on a yellow bin medication and any of the dependent variables.

Highly significant correlations were found between the number of all drugs taken and numbers of medical visits (*r*=+0.49; *P*<0.001) and healthcare visits (*r*=+0.35; *P*=0.0005) (Table 1). An average of two of the 26 interpretive report panel drugs were being taken at the end of the study year by the nine red bin status subjects, and this exceeded (*P*=0.002) the average number of panel drugs taken by green (1.2) or yellow (1.4) status subjects. A similar trend was observed for the number of all psychiatric drugs (that is, panel and nonpanel drugs) taken by red bin subjects (2.7) versus green (1.9; *P*=0.044) or yellow (2; *P*=0.084) drug bin status subjects. The number of all drugs taken by subjects was the same for those in the red (6.4), green (6.3) and yellow (5.9) bins. There was no correlation between the number of panel or all psychiatric drugs taken and any of the eight dependent measures (Table 1).

**Figure 5:**
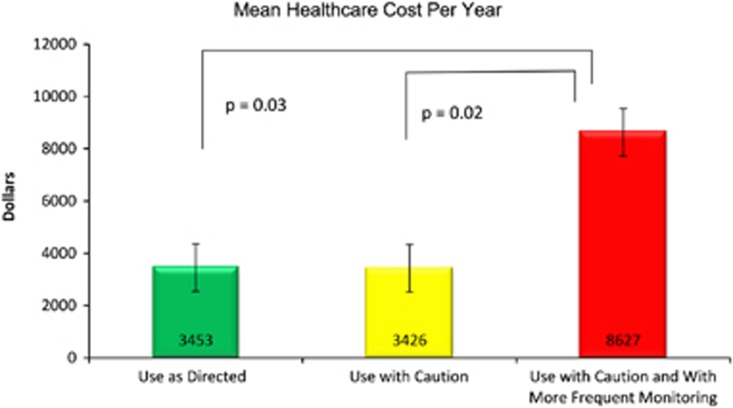
Calculated healthcare spend (2010 dollars) for the patients whose psychiatric GeneSight panel drug prescription(s) were in the ‘use as directed' (*n*=40) category, or had one or more ‘use with caution' (*n*=48) or ‘use with caution and more frequent monitoring' (*n*=9) drug ranked as the most severe category among the panel drug(s) they were prescribed. Significantly greater healthcare spends were calculated for the nine red-bin status patients than those in the green or yellow categories (*t*-test).

The mean healthcare utilization cost calculated for red bin subjects was higher at $8627, compared with $3453 calculated for green bin subjects (*P*=0.024) and $3426 for yellow bin subjects. (*P*=0.027, Figure 5) yielding an average annual increase in healthcare cost of $5188 for red bin subjects.

## Discussion

A goal of pharmacogenomic-based personalized medicine is to provide information that can better define treatments for individual patients and increase the rate or amount of their therapeutic improvement. In addition to, and possibly as a result of these clinical benefits, pharmacogenomic testing also has the potential to decrease direct and indirect medical costs. Chou *et al*^11^ demonstrated increased healthcare costs in CYP2D6 poor and ultrarapid metabolizers due to longer hospitalizations and increased adverse events in these patients.

The present retrospective analysis is consistent with and expands upon these earlier results, by showing that some of the economic healthcare burden for psychiatric patients may be predicted by a multi-gene, pharmacogenomic approach. Specifically, subjects whose medication status was identified by the binning system of the GeneSight report to be in the ‘use with caution and more frequent monitoring' (red bin) category had the highest numbers of medical visits, total healthcare visits, medical absence days and disability claims compared with all other subjects. Subjects taking red bin medications had nearly twice the number of healthcare visits and over four times the average number of disability claims during the retrospective study year.

The results further suggest that both medical and productivity cost savings may be obtained prospectively if the psychiatric pharmacogenomic test is used to guide psychotropic medication selection, that is, shifting patients away from drugs that are found to be in the red bin category. Improved psychotropic medication selection could, for example, provide a ‘pharmacoeconomic' benefit for an individual with a cytochrome P450 2D6 genotype that renders the enzyme unable to metabolize an SSRI (Figure 2). This patient may experience medication toxicity due to excessive SSRI blood levels, enhanced side effects, poor outcome and increased healthcare costs.^18, 19^ This problem could be exacerbated when concomitant psychotropic and nonpsychotropic medications increase the variability of drug metabolism, which may explain the correlation we obtained between the numbers of all medications and the numbers of healthcare visits and medical visits.

Consistent with the present results, lower antidepressant efficacy was found previously in patients whose medications were in the red bin versus those in the green and yellow bin category.^9, 10^ In these prospective studies, GeneSight pharmacogenomic test results were generated for all patients at the initiation of the study, while patients in the treatment-as-usual control arm were given their results only after the study was completed. Pharmacogenomic-directed prescribing reduced the incidence of adverse drug reactions and improved the efficacy of antidepressant medication regimens, particularly in patients who were taking red bin medications at the beginning of the studies.^9, 10^ Additional studies will be needed to confirm these and related pharmacoeconomic outcomes for depressed, anxious and other patients who most commonly use the medications on the GeneSight panel (Figure 2).

One potential limitation of the study was that red bin status was associated with a higher number of psychiatric medications, and that this was linked. However, the lack of correlation between any of the eight dependent measures and the number of either prescribed panel drugs or all psychiatric drugs, indicates that the pharmacogenomic-determined red bin status, and not the numbers of psychiatric drugs taken, was associated with increases in healthcare utilizations. Interestingly, healthcare visits and medical visits did correlate positively with the number of all drugs taken, demonstrating the sensitivity of this method to identify such covariates (Table 1).

Other limitations include the retrospective nature of the study, the heterogeneity of subject diagnoses and the relatively small sample. The UHS contracts with only one psychiatrist, thus, all patients were under her care. Additionally, because billing and reimbursement data were not available from the study site, they were imputed from available sources.

The finding that the number of all prescribed medications correlated positively with two outcomes (overall healthcare visits and medical visits), shows that a greater healthcare burden was associated with overall prescription number, more so than when psychiatric drugs alone are considered. The problematic nature of drugs in the red bin category derives from their more aberrant metabolism, efficacy or both based on individual pharmacokinetic or pharmacodynamic polymorphisms. Thus, it appears that the GeneSight test adds qualitative dimensions beyond simple prescription numbers to improve the accuracy of predicting healthcare burden.

In conclusion, a six gene, pharmacogenomic-based interpretive test identified significant increases in healthcare utilization and estimated greater costs ($5188) for individual psychiatric patients whose medication status for a year was identified as having been suboptimal. This observation reflects the ability of an integrated, multi-gene analysis of individual patient polymorphisms to predict variations in psychotropic drug metabolism and response, and was independent of the number of prescribed psychiatric medications. The study results demonstrate that patients on more problematic red bin drugs have increased direct and indirect medical utilization, and suggest that switching patients off of these medications may decrease medical utilization.

These and prior results^9, 10^ demonstrate the potential predictive ability of the GeneSight test and its interpretive report to help improve clinical outcomes, while reducing costs associated with increased medical utilization for patients taking inappropriate psychotropic medications. This is the first study in psychiatry of the predictive value of multi-gene pharmacogenomic information on healthcare utilization, and the first to demonstrate statistically significant predictions for psychiatry of health utilization based on genotype. The cost savings results compare favorably with those of Chou and colleagues,^11^ who estimated a $4000–6000 greater cost in treating CYP2D6 ultrarapid metabolizer/poor metabolizer psychiatric patients than extensive metabolizer/intermediate metabolizer patients. Additional and larger prospective studies with diverse patient populations will be of value in demonstrating the economic benefits and clinical utility of this approach, and broadening its acceptance in routine clinical practice.

## Figures and Tables

**Table 1:** No correlation between number of panel or psychiatric drugs and healthcare outcomes that are predicted by GeneSight drug status

*Outcome measured (number of events/year)*	*Statistic*	*Numbers of drugs taken by patients*			*Increase healthcare burden for patients on red bin drugs?*
		*Panel drugs*	*Psychiatric drugs*	*All drugs*	
Healthcare visits	*r*	−0.04	0.11	0.26	
	*P*	0.68	0.29	0.01	Yes
	F	0.23	1.13	6.98	
Medical visits	*r*	−0.11	0.05	0.40	
	*P*	0.30	0.66	<0.0001	Yes
	F	0.04	0.10	18.83	
Psychiatric visits	*r*	0.05	0.12	−0.02	
	*P*	0.63	0.24	0.87	trend; ns
	F	1.05	1.18	0.01	
ER visits	*r*	−0.04	0.08	0.05	
	*P*	0.70	0.42	0.62	No
	F	0.15	0.65	0.25	
Hospitalizations	*r*	−0.07	0.11	0.13	
	*P*	0.50	0.28	0.20	No
	F	0.45	1.18	1.69	
Days in hospital	*r*	−0.05	−0.27	0.004	
	*P*	0.87	0.36	0.99	No
	F	0.03	0.92	0.0002	
Days off work	*r*	0.08	−0.04	−0.05	
	*P*	0.45	0.73	0.64	trend; ns
	F	0.59	0.12	0.21	
Disability claims	*r*	0.002	−0.02	0.005	
	*P*	0.98	0.85	0.96	Yes
	F	0.0005	0.03	0.002	

Abbreviations: ER, emergency room; ns, not significant.

*r*=correlation coefficient; *P*=significance value for correlation coefficient; F=ANOVA value.

## Notes

Genetic testing and funding for data collection and analysis personnel were provided by AssureRx Health, Inc. JW, JD Allen and C Anthony Altar, are employed by AssureRx Health, Inc. The principal investigator, A Spahic-Mihajlovic, has served as a speaker for AssureRx Health.
